# From recurrent flank pain to systemic oxalosis: a case of primary hyperoxaluria type 1 requiring dual organ transplantation

**DOI:** 10.1097/MS9.0000000000005158

**Published:** 2026-05-07

**Authors:** Syed Muhammad Salman Hassan, Nizra Amjad, Mugheesa Rab, Ayesha Malik, Ayesha Jamal, Momal Fatima, Muhammad Hasnain Sadiq, Aleeza Khan, Muddassir Khalid

**Affiliations:** a Department of Medicine, Nishtar Medical University, Multan, Pakistan; b Department of Medicine, Rawalpindi Medical University, Rawalpindi, Pakistan

**Keywords:** AGXT gene, end-stage renal disease, liver-kidney transplant, primary hyperoxaluria, systemic oxalosis

## Abstract

**Introduction and importance:**

Primary hyperoxalurias (PH) are rare autosomal recessive inherited disorders that disrupt the metabolism of glyoxylate and oxalate. The most common type, primary hyperoxaluria type 1 (PH1), is caused by a deficiency in the liver enzyme alanine-glyoxylate aminotransferase (AGT), leading to the overproduction and excessive urinary excretion of oxalate.

**Case presentation:**

On routine evaluation, the patient presented with deranged renal function and echogenic kidneys. Imaging via KUB X-ray revealed radio-opaque kidney stones, small shrunken kidneys, and nephrocalcinosis, indicating progression to end-stage kidney disease (ESKD). Additional systemic manifestations included increased lumbar bone density and pulmonary fibrosis. Diagnosis was definitively confirmed through elevated 24-hour urinary oxalate levels and genetic screening showing an *AGXT* gene mutation. This necessitated combined liver–kidney transplantation (CLKT): the liver graft provides the missing AGT enzyme to stop oxalate production, while the kidney graft replaces damaged organs and discontinues chronic dialysis.

**Clinical discussion:**

PH1 must be suspected in pediatric patients presenting with recurrent urolithiasis or nephrocalcinosis, especially in clinical cases of consanguinity. Early interventions – high fluid intake, crystallization inhibitors, and pyridoxine – can help preserve kidney function. For patients reaching ESKD, combined transplantation is the most effective approach to correct the underlying metabolic error and stop oxalate accumulation.

**Conclusion:**

This case highlights the diagnostic challenge of PH1, where the initial presentation with flank pain and urinary symptoms may mimic a urinary tract infection, placing the patient at potential risk of urosepsis if misdiagnosed or untreated. CLKT remains the definitive treatment in advanced disease.

## Introduction

Primary hyperoxaluria type 1 (PH1), the most severe form of hyperoxaluria, is a rare genetic disorder caused by mutations in the AGXT gene, resulting in a deficiency of the enzyme alanine-glyoxylate aminotransferase (AGT). The enzymatic defect leads to glyoxylate accumulation, which is subsequently converted into oxalate, resulting in crystalluria, nephrolithiasis, nephrocalcinosis, and progressive kidney injury. PH1 can present in infancy or early childhood with nephrocalcinosis or chronic kidney disease (CKD), or later in life with recurrent kidney stones. Nephrolithiasis and nephrocalcinosis are present in 90 and 48% of cases at the time of diagnosis, respectively. About 20% of patients are diagnosed after reaching end-stage kidney disease (ESKD), and 7% are diagnosed after disease recurrence post-kidney transplantation^[^1^]^.

HIGHLIGHTS

- Primary hyperoxaluria type 1 is a rare cause of end-stage renal disease and systemic oxalosis.
- Early diagnosis and pyridoxine-based therapy can delay disease progression.
- Genetic analysis identifies more than 140 mutations linked to disease severity.
- Isolated kidney transplant carries the risk of recurrence and graft loss.
- Combined liver–kidney transplant offers the best survival and metabolic cure.

PH1 has an estimated prevalence of 1–3 cases per million in Europe and North America, with higher rates observed in consanguineous populations, such as those in the Middle East and North Africa. Around 20–25% of affected individuals progress to kidney failure by a median age of 25 years, and up to 16% require combined liver–kidney transplantation (CLKT). Despite advancements in treatment, there remains a substantial gap in understanding the systemic complications, which impact up to 34.6% of patients, and healthcare resource usage, with over 80% requiring hospitalization or emergency care^[^2^]^.

CLKT is the most effective curative treatment for ESKD in PH1, addressing both the enzymatic defect in the liver and the irreversible kidney damage caused by systemic oxalate accumulation. While CLKT significantly improves outcomes compared to isolated transplants, challenges remain, as diagnosis is often delayed until ESKD or post-transplant complications arise. Additionally, a limited understanding of the genotype-phenotype correlations in PH1 further complicates disease management^[^3^]^.

This case highlights the importance of early diagnosis through genetic testing and multidisciplinary management, demonstrating the critical role of CLKT in preventing systemic complications and improving survival in patients with severe PH1.

This case report has been prepared in line with the CARE checklist.

## Case history

An 18-year-old boy presented to the medical out patient department with complaints of flank pain, fever, and burning micturition. On initial clinical evaluation, a urinary tract infection (UTI) was suspected, and the patient was started on conservative management. Routine investigations, including a complete urine examination and renal function tests, were ordered.

**Figure 1.**
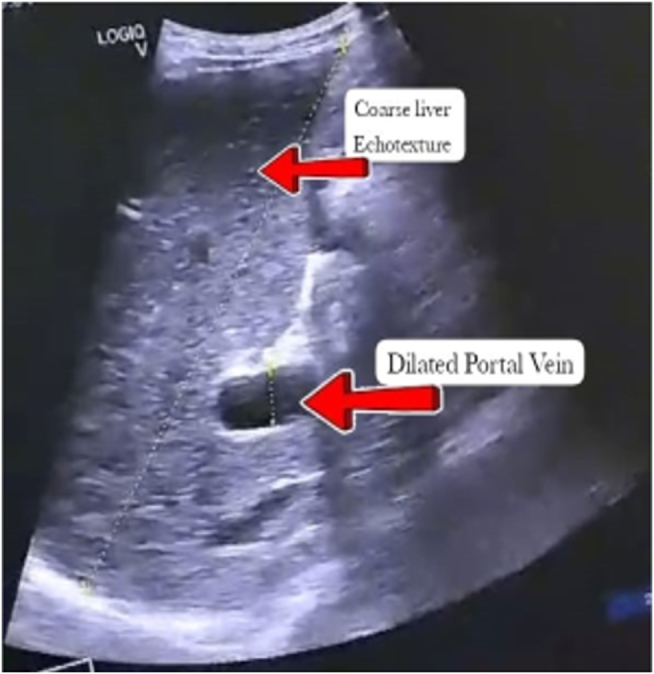
Ultrasonography showing coarse liver texture and a dilated portal vein.

**Figure 2.**
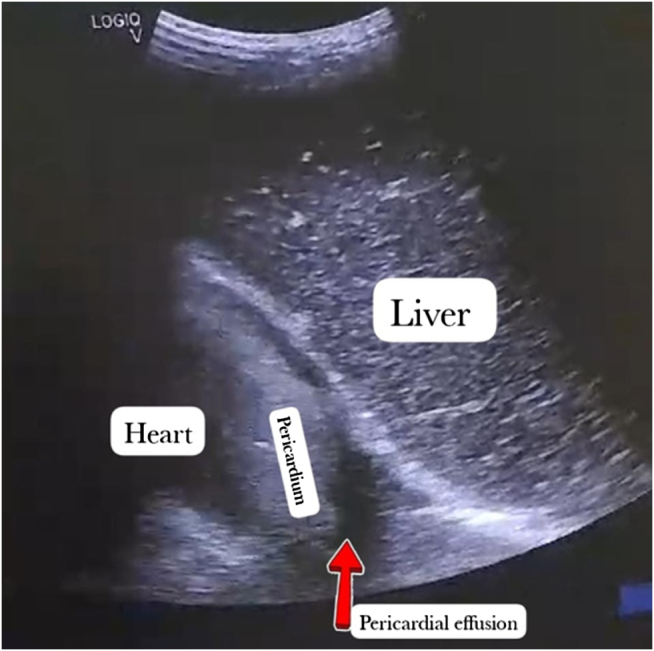
Ultrasonography showing pericardial effusion.

The patient’s renal parameters were significantly deranged, with markedly elevated creatinine levels. However, his complete urine examination showed no evidence of pus cells, red blood cells, or white blood cell casts. Additionally, urine culture and sensitivity results were negative. To rule out nephrolithiasis, an abdominal ultrasound was performed, which revealed no kidney stones (Figs 1 and 2). The absence of visible calculi on ultrasonography may be attributed to the presence of diffuse nephrocalcinosis and markedly echogenic renal parenchyma, which can obscure smaller calculi and reduce the sensitivity of ultrasound in detecting stones in advanced CKD.

Further imaging with an X-ray provided crucial findings, showing the presence of kidney stones in both kidneys, nephrocalcinosis, and small, shrunken kidneys – findings suggestive of ESKD. Additionally, there was evidence of increased bone density in the lumbar vertebrae, fractures with compression deformities, and interstitial lung fibrosis.

**Figure 3.**
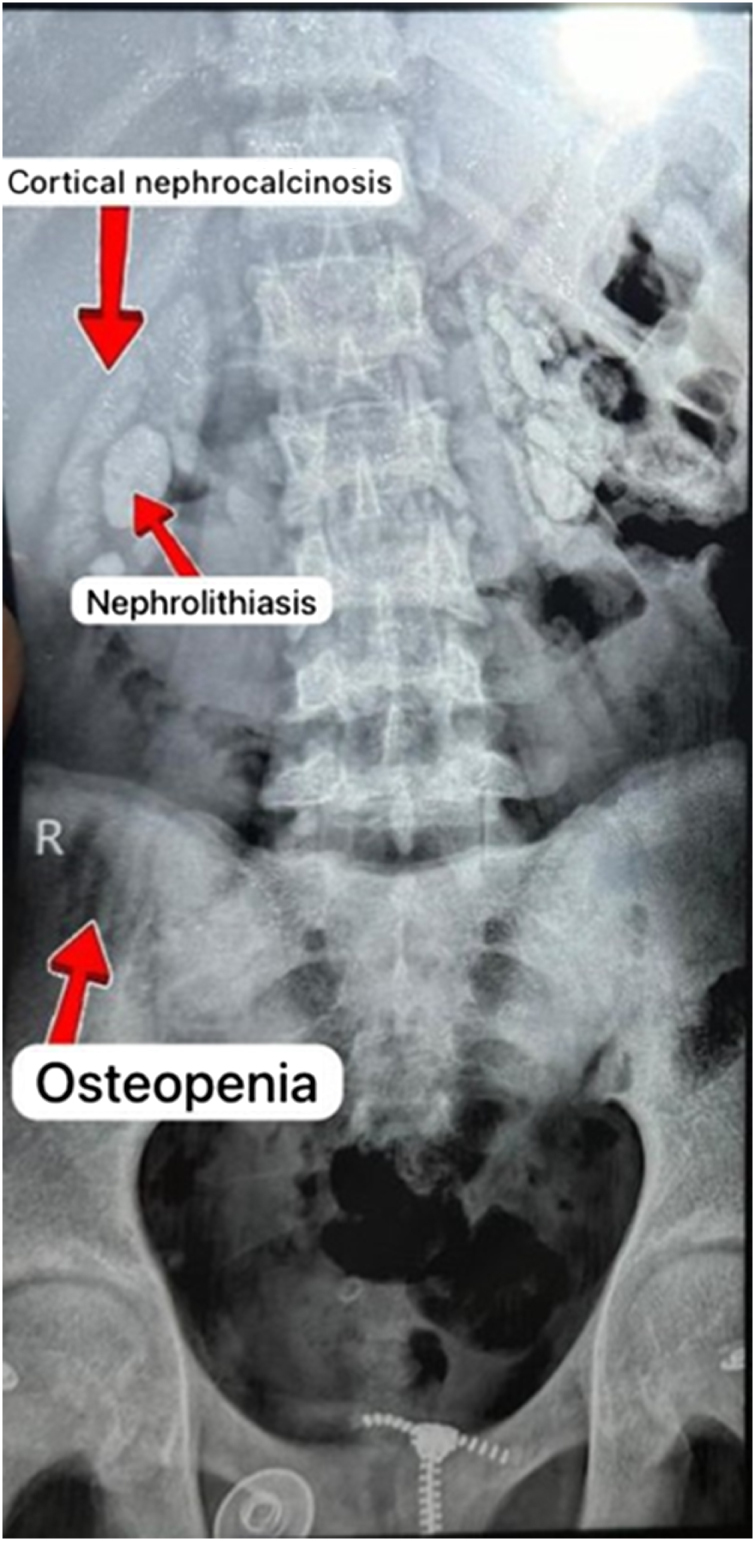
X-ray showing evidence of osteopenia.

**Figure 4.**
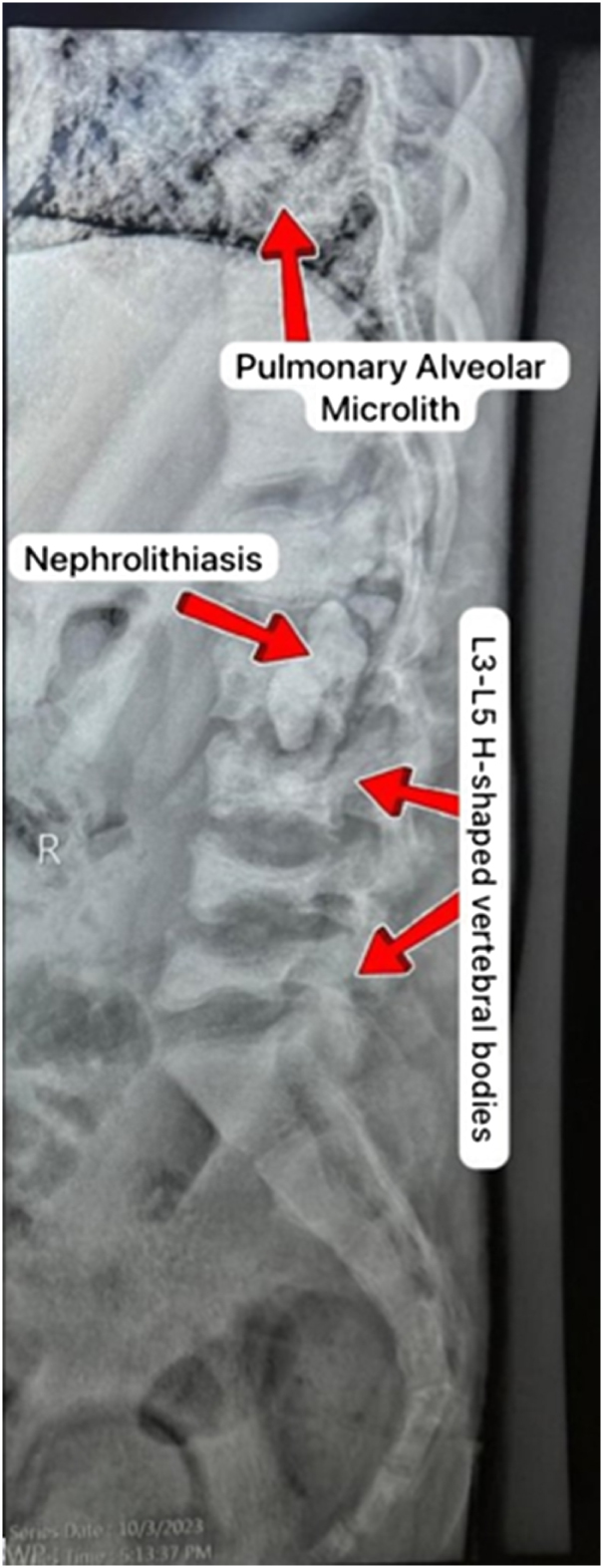
X-ray showing pulmonary alveolar microlith, nephrolithiasis, and L3–L5 H-shaped vertebral bodies.

The patient had already been undergoing dialysis via an arteriovenous fistula in his right arm and was initially being evaluated for a liver transplant. This initial consideration for liver transplantation was based on unexplained systemic features, including progressive renal failure, skeletal changes, and suspected metabolic dysfunction. However, a definitive diagnosis had not yet been established at that time. Abdominal ultrasound findings included an enlarged spleen, mild liver parenchymal changes, and highly echogenic kidneys, further supporting the diagnosis of CKD (Figs 3 and 4).

Given the atypical presentation, further investigations were conducted, leading to the diagnosis of PH1 – a rare genetic disorder caused by a mutation in the AGXT gene, resulting in excessive oxalate production. Genetic testing confirmed a homozygous pathogenic splice-site variant in the AGXT gene (NM_000030.2 c.596-2A>G), with no clinically relevant copy number variations identified, establishing the definitive genetic diagnosis of autosomal recessive PH1. Unfortunately, his condition had progressed to the point where he required both a liver and a kidney transplant. A new liver transplant would provide the missing enzyme and help mitigate systemic oxalosis, potentially preventing further kidney damage, but he needed both organs to be transplanted.

The patient subsequently underwent simultaneous CLKT. Post-transplant immunosuppression followed the standard protocol used for dual-organ recipients at our center. Induction therapy consisted of basiliximab (an anti-IL-2 receptor monoclonal antibody), administered as 20 mg intravenously on postoperative days 0 and 4. Maintenance immunosuppression included tacrolimus, mycophenolate mofetil (MMF), and corticosteroids. Tacrolimus was initiated at 0.05 mg/kg twice daily, with doses adjusted to maintain trough levels of 8–10 ng/mL during the first 3 months after transplantation and 5–8 ng/mL thereafter. Drug levels were monitored closely due to the potential risk of calcineurin inhibitor–related nephrotoxicity in the renal allograft. MMF was administered at 500 mg twice daily. Corticosteroid therapy was gradually tapered from an intraoperative dose of methylprednisolone (500 mg IV) to oral prednisolone, which was discontinued by the end of the third postoperative month. The patient tolerated the regimen well, and no episodes of acute rejection were observed during the follow-up period. Although the patient did not have overt hepatic failure, the MELD score was not significantly elevated, which is consistent with PH1, where liver function is typically preserved despite the underlying enzymatic defect. Exception criteria were therefore applied to prioritize transplantation.

Following the diagnosis, the patient underwent a successful simultaneous CLKT, which resulted in stabilization of renal function and resolution of dialysis dependence. The patient was successfully liberated from dialysis and discharged on a standard immunosuppressive regimen, with outpatient follow-up arranged. Long-term monitoring for systemic oxalate clearance and extra-renal manifestation resolution was planned; however, extended follow-up data are not available at the time of this report. Only short-term post-transplant outcomes were available at the time of reporting, which represents a limitation of this case.

## Discussion

PH1 is one of the rarest yet essential causes of end-stage renal disease (ESRD). Symptoms of this disease are secondary to urolithiasis, which may lead to acute kidney injury or cause a UTI^[^4^]^. Early diagnosis is essential for treating patients with recurrent nephrolithiasis and renal stones. Conservative treatment, including fluid therapy, vitamin B6 administration, and urine alkalization, must be initiated once the initial work-up is complete^[^5^]^. Research indicates that pyridoxine hydrochloride can lead to a reduction in urinary oxalate levels in about 30% of patients with PH. However, the exact reasons for this response remain unclear^[^6,7^]^ Alkalinizing the urine with alkali citrate can help lower urinary calcium oxalate saturation by forming complexes with calcium, which may inhibit stone formation or nephrocalcinosis^[^8^]^. Limiting oxalate intake has limited effectiveness since the primary source of oxalate is produced internally, and patients with PH absorb less oxalate from their intestines compared to healthy individuals^[^9^]^. While these strategies can help in slowing down the disease progression, in advanced cases or a patient with ESRD, this mode of treatment is not of much help.

Recent advances in molecular therapy have significantly changed the management of PH1. RNA interference (RNAi) therapies, particularly lumasiran, have emerged as important treatment options for reducing oxalate production. Lumasiran is a subcutaneous small-interfering RNA that targets hepatic glycolate oxidase (encoded by *HAO1*), thereby decreasing the conversion of glyoxylate to oxalate. It received approval from both the FDA and the European Medicines Agency in 2020 for use in patients of all age groups. In the pivotal ILLUMINATE-A trial, treatment with lumasiran resulted in a 65.4% reduction in urinary oxalate excretion, with most patients achieving normal or near-normal oxalate levels after 6 months of therapy^[^10^].^ Similarly, the ILLUMINATE-B phase III study in infants and young children demonstrated a 72% reduction in the urinary oxalate-to-creatinine ratio after 6 months, supporting the safety and effectiveness of RNAi therapy even in very young patients^[^11^]^

Another RNAi agent, nedosiran, which targets hepatic lactate dehydrogenase A (LDHA), received FDA approval in 2023 for patients aged 9 years and older with PH1 who have relatively preserved kidney function (eGFR ≥30 mL/min/1.73 m^2^)^[^12,13^]^. These therapies represent a major advancement in the treatment of early-to-moderate stage PH1. They can significantly reduce oxalate production, potentially slowing or preventing disease progression when started early. However, these agents are unable to reverse established ESKD or rapidly eliminate the large systemic oxalate burden that accumulates over years of disease progression.

In the present case, the patient had already progressed to ESKD, with imaging demonstrating small, shrunken kidneys as well as evidence of systemic oxalosis affecting the skeletal and pulmonary systems. At this stage of the disease, RNAi therapy alone would not be sufficient to restore kidney function or adequately clear the accumulated oxalate stores. Therefore, although RNAi therapies such as lumasiran have transformed the management of early PH1, they do not replace the need for transplantation in advanced disease. CLKT was therefore pursued as the most definitive treatment option, correcting the underlying enzymatic defect through the liver graft while simultaneously replacing the non-functional kidneys, ultimately providing the best opportunity for long-term survival and systemic oxalate clearance.

According to data obtained from the US Renal Data System (USRDS), the projected survival rate for a patient without a transplant is 40% at 5 years and 20% at 9 years after the establishment of a definitive diagnosis^[^14^]^. Patients with diagnosed PH1 have markedly increased excretion of oxalate greater than 1 mmol/1.73 m². In some patients, it may exceed 1.5–3 mmol (normal oxalate excretion is 0.5 mmol/1.73 m² per day)^[^15^]^. In renal insufficiency, the kidney’s potential capacity to excrete oxalate is reduced. Many patients with ESRD may be anuric, which can increase plasma oxalate concentration, aiding in the diagnosis of PH1, but does not confirm it, as isolated renal insufficiency can also result in such findings.

Molecular genetic analysis is now a mainstay of diagnosis, with its usage increasing over the past few years. To date, 146 mutations have been described that are associated with PH1^[^16^]^. The identified splice-site variant (c.596-2A>G) disrupts normal AGXT mRNA splicing, resulting in the complete loss of functional AGT enzyme. This is consistent with the severe phenotype and rapid progression to ESKD seen in our patient, and, unlike missense variants such as p.Gly170Arg, this mutation is unlikely to carry any meaningful response to pyridoxine supplementation. As the AGXT gene is relatively small, complete genomic sequencing and identification of mutations are relatively easy and readily available options to detect both common and rare mutations^[^16^]^.

Accumulation of oxalate levels in the body is the primary cause of long-term complications^[^17^]^. The oxalate accumulation in extra-renal organs causes livedo reticularis, digital infarcts, osteoarthritis, pancytopenia, and cardiovascular diseases, leading to death^[^18,19^]^.

Isolated kidney transplants can enhance oxalate clearance but are limited due to recurrence and allograft loss. The European Dialysis and Transplant Association showed poor outcomes for patients with isolated renal transplants in the 1980s, with 3-year patient and graft survival rates of 74% and 20%, respectively. However, survival rates have improved due to early detection and immunosuppressive therapy^[^20,21^]^. Data from the USRDS registry also show a significant increase in the 8-year survival rate with liver-kidney transplants, which stands at 76% compared to isolated kidney transplants, which stand at 47.9%^[^22^]^.

Combination transplants can be given simultaneously or one-by-one, with simultaneous transplants providing immediate relief from metabolic abnormalities^[^4^]^.

One series of simultaneous kidney and liver transplants demonstrated a decrease in renal function after 3, 6, and 12 months post-transplant in the pediatric age group in patients with PH1 as their established diagnosis, compared to those with other diagnoses^[^22^]^.

According to data from the American Association for the Study of Liver Diseases, a liver transplant is indicated for any acute or chronic liver failure^[^23^]^. Despite the presence of normal liver function, PH is considered an indication for a liver transplant. The need for a hepatic transplant is calculated using a clinical score called the MELD score, which takes into consideration serum creatinine, serum bilirubin, and the international normalized ratio^[^23^]^. Patients with PH1 have a normal MELD score, indicating a 90–97% three-month survival rate. In 2006, exceptions were made to accommodate patients with AGT enzyme deficiency, as the score does not portray systemic oxalosis or transplant urgency^[^24^]^. Patients over 1 year old with ESRD risk are assigned a MELD score, indicating a 15% risk with ESRD or a 10% risk without it, with an additional 10% risk added every 3 months. Given the variety of symptoms and the disparity in the scoring system for assigning priority for transplantation, consideration should be given to a separate scoring system for determining the need for urgent transplantation, with multi-organ damage due to systemic oxalosis serving as an essential criterion.

This case underscores the importance of considering rare metabolic causes, such as PH1, in young patients presenting with recurrent flank pain and renal dysfunction, particularly when initial investigations are inconclusive. Early recognition can prevent progression to systemic oxalosis and avoid the need for complex dual-organ transplantation.

## Conclusion

PH1 is an uncommon genetic condition marked by the overproduction of oxalate, which can result in end-stage renal failure. In this instance, we treated a child with the AGXT mutation who required both kidney and liver transplants. PH1 is a rare but severe metabolic disorder that can lead to progressive renal failure and systemic oxalosis if not diagnosed early. This case highlights the consequences of delayed recognition, where a young patient progressed to ESKD requiring CLKT. Early metabolic and genetic evaluation in patients with unexplained nephrolithiasis or renal dysfunction is critical to prevent irreversible complications. A multidisciplinary approach is essential to optimize outcomes in PH1.

TITAN guidelines: This manuscript complies with TITAN Guidelines 2025, declaring no use of artificial intelligence^[^25^]^.

## Data Availability

Data available on request from the authors.
